# Does the intramedullary femoral canal plug reduce blood loss during total knee arthroplasty?

**DOI:** 10.1186/s43019-022-00160-4

**Published:** 2022-06-28

**Authors:** Yutthana Khanasuk, Srihatach Ngarmukos, Aree Tanavalee

**Affiliations:** 1 Department of Orthopedics, Queen Savang Vadhana Memorial Hospital, 290 Jermjomphon Road, Sri-Racha, Chonburi, 20110 Thailand; 2 Department of Orthopedics, Faculty of Medicine, Chulalongkorn University, 1873 Rama IV Road, Pathumwan, Bangkok, 10330 Thailand

**Keywords:** Plug, Seal, Blood loss, Total knee arthroplasty, Total knee replacement, TKA

## Abstract

**Introduction:**

The benefit of the femoral canal bone plug during total knee arthroplasty (TKA) in reducing blood loss has never been proven. The aim of this meta-analysis was to determine whether the femoral canal bone plug significantly reduces blood loss in primary TKA.

**Method:**

All studies published before December 2021 were searched. The inclusion criteria were randomized controlled trials comparing blood loss between TKA with plugged and unplugged femoral intramedullary canal, respectively. The primary outcome was postoperative hemoglobin reduction.

**Results:**

Five studies with a total of 717 patients (361 in the plugged group, 356 in the unplugged group) met the criteria for inclusion in the meta-analysis. The mean difference in hemoglobin level between the two groups was 0.92 g/dL, with significantly less hemoglobin reduction in the plugged group (95% confidence interval [CI] − 1.64 to − 0.21, *p* = 0.01). The patients in the plugged group also had a significantly lower risk of receiving a blood transfusion (risk ratio 0.58, 95% CI 0.47–0.73, *p* < 0.00001).

**Conclusions:**

This meta-analysis demonstrates that using a femoral canal bone plug can significantly reduce blood loss and lower the risk ratio of blood transfusion in patients undergoing TKA.

## Introduction

Total knee arthroplasty (TKA) is associated with the potential for postoperative blood loss due to extensive soft tissue dissection, uncovered metaphyseal bone cuts, and neglected small vessel injuries [1]. Several studies have reported total blood loss ranging from 600 to 1790 mL and a postoperative transfusion rate of 11–21% [2–4]. As in any modern surgery, blood transfusion should be avoided because it might cause rare but severe complications, such as immunological reactions, infections, hemolysis, renal failure, and death [5, 6].

The conventional TKA technique requires drilling of the femoral intramedullary canal for insertion of an intramedullary cutting guide, which is a potential source of blood loss through the opened medullary hole. It was believed that sealing the drill hole with a bone plug or bone cement could help reduce blood loss, hence reducing the need for blood transfusion.

Several studies have compared the amount of blood loss between TKA with and without femoral canal bone plug, but the results remain equivocal [4, 7–10]. Here we report the results of a meta-analysis aimed to verify the efficacy of the femoral canal bone plug in reducing blood loss and transfusion rate in TKA.

## Methods

### Search strategy

This study was performed in accordance with the principles of the Declaration of Helsinki. Approval was granted by the Ethics Committee of Queen Savang Vadhana Memorial Hospital, The Thai Red Cross Society No. 005/2022. This study was registered at the International Database of Prospectively Registered Systematic Reviews in Health and Social Care (PROSPERO), identification no. CRD42016045758. No informed consent was obtained from the participants included in the study.

We searched the PubMed, Scopus, Cochrane Library, Ovid, EMBASE databases, as well as offline sources, for relevant studies published before December 2021. The search terms were “plug,” “seal,” “total knee replacement,” and “total knee arthroplasty.” We also searched unpublished and ongoing studies in the clinicaltrials.gov and PROSPERO databases for investigations that had similar or different surgical or perioperative protocols but which could affect blood loss.

Inclusion criteria were randomized controlled trials (RCTs) that compared the amount of blood loss in TKA between patients with and without femoral canal bone plugs (‘plugged’ and ‘unplugged’ groups, respectively). Studies without a control group, studies with incomplete data, and retrospective studies were excluded.

The primary outcome was postoperative hemoglobin reduction. The secondary outcomes were blood transfusion rate, total drained blood, and drained blood at 24 h after surgery.

### Study identification and selection

Two authors (YK and SN) independently reviewed study identification and selection. Any disagreement was discussed and resolved. Cochrane Reviewers’ Handbook [11] was applied to assess the methodological qualities. Included studies are described in Table 1.

**Table 1:** Studies Included for the analysis

Study	Total no. of patients	No. of female patients	Total knee arthroplasty		Outcomes	Results
			With femoral canal bone plug (*n*)	Without femoral canal bone plug (*n*)		
Ko et al. 2003 [7]	262	198	128	134	- Hemoglobin reduction - Total drained blood - Drained blood at 24 h postoperatively - Transfusion rate	Bone plug effectively reduced blood loss
Kumar et al. 2000 [8]	120	74	65	55	- Total drained blood - Drained blood at 24 h postoperatively	Bone plug was effective in reducing blood loss
Torres-Claramunt et al. 2014 [9]	134^a^	96	67	67	- Hemoglobin reduction - Total drained blood - Drained blood at 24 h postoperatively - Transfusion rate	Bone plug had no effect on blood loss or transfusion rate
Li et al. 2017 [4]	120	91	60	60	- Hemoglobin reduction, - Total drained blood, - Transfusion rate	Bone plug significantly reduced blood loss and transfusion rate
Tanasubsinn et al. 2017 [10]	81	61	41	40	- Total drained blood - Transfusion rate	Bone plug did not reduce either blood loss or transfusion rate

^a^Torres-Claramunt [9] reported 201 patients who were allocated to 3 groups: with bone plug, with bone cement, and without bone plug, respectively. Only data on the groups receiving/not receiving bone plug were included here

### Statistical analysis

Data analysis was performed with RevMan version 5.0.22 (The Nordic Cochrane Centre, Copenhagen, Denmark). Statistical heterogeneities of all outcomes were assessed with the Chi-square test and *I*^2^ test. A fixed-effects model was used when the *p* value was ≥ 0.1 and the *I*^2^ value was ≤ 50% (*I*^2^ between 0 and 25% = no statistical heterogeneity, *I*^2^ between 25.1 and 50% = low statistical heterogeneity). A random-effects model was used when the *p*-value was < 0.1 and the *I*^2^ value was > 50% (*I*^2^ between 50.1–75% = moderate statistical heterogeneity, *I*^2^ between 75.1 and 100% = high statistical heterogeneity).

## Results

### Study identifications

**Fig. 1:**
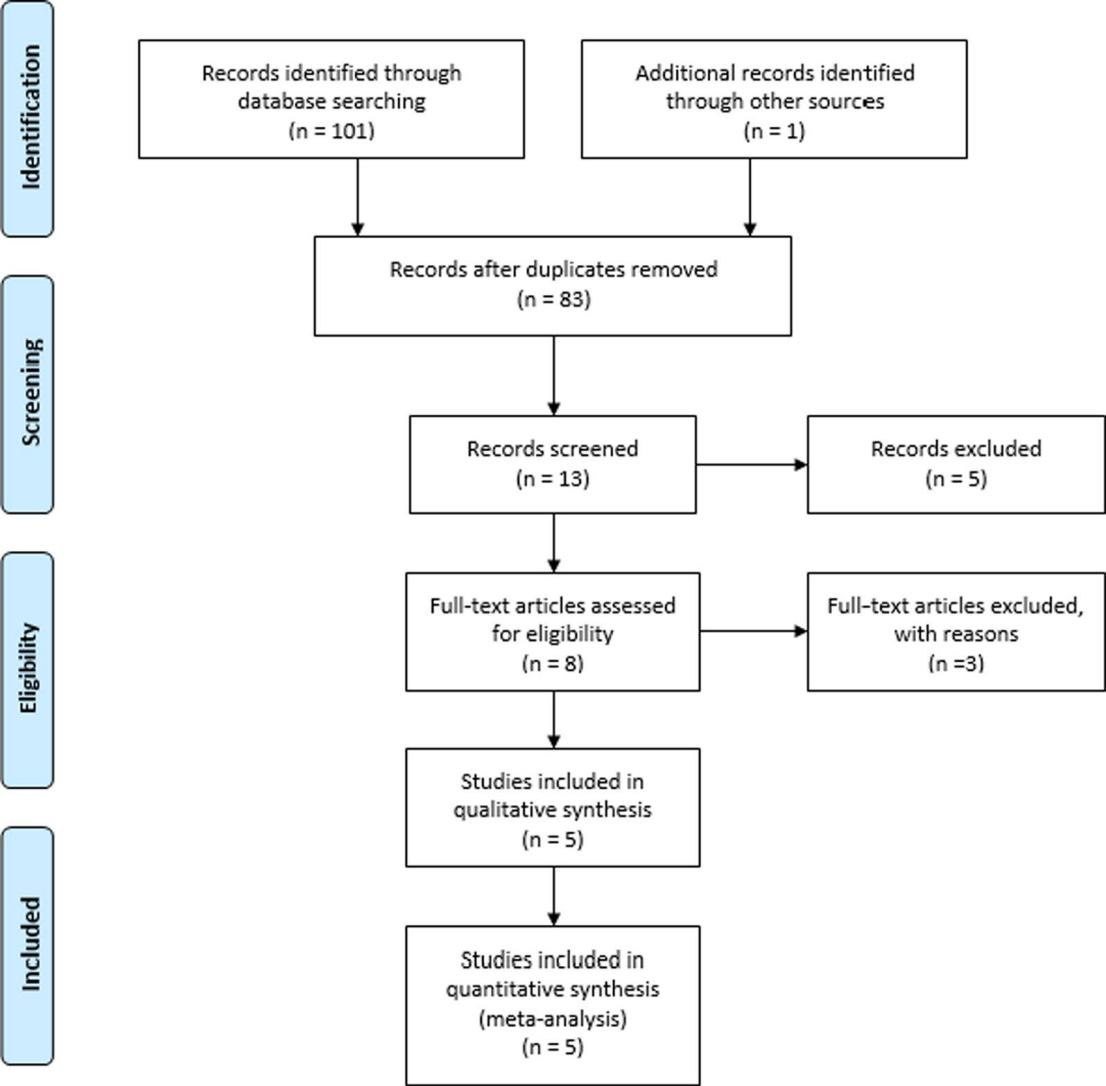
PRISMA (Preferred Reporting Items for Systematic Reviews and Meta-Analyses) flow diagram summarizing study selection process

**Fig. 2:**
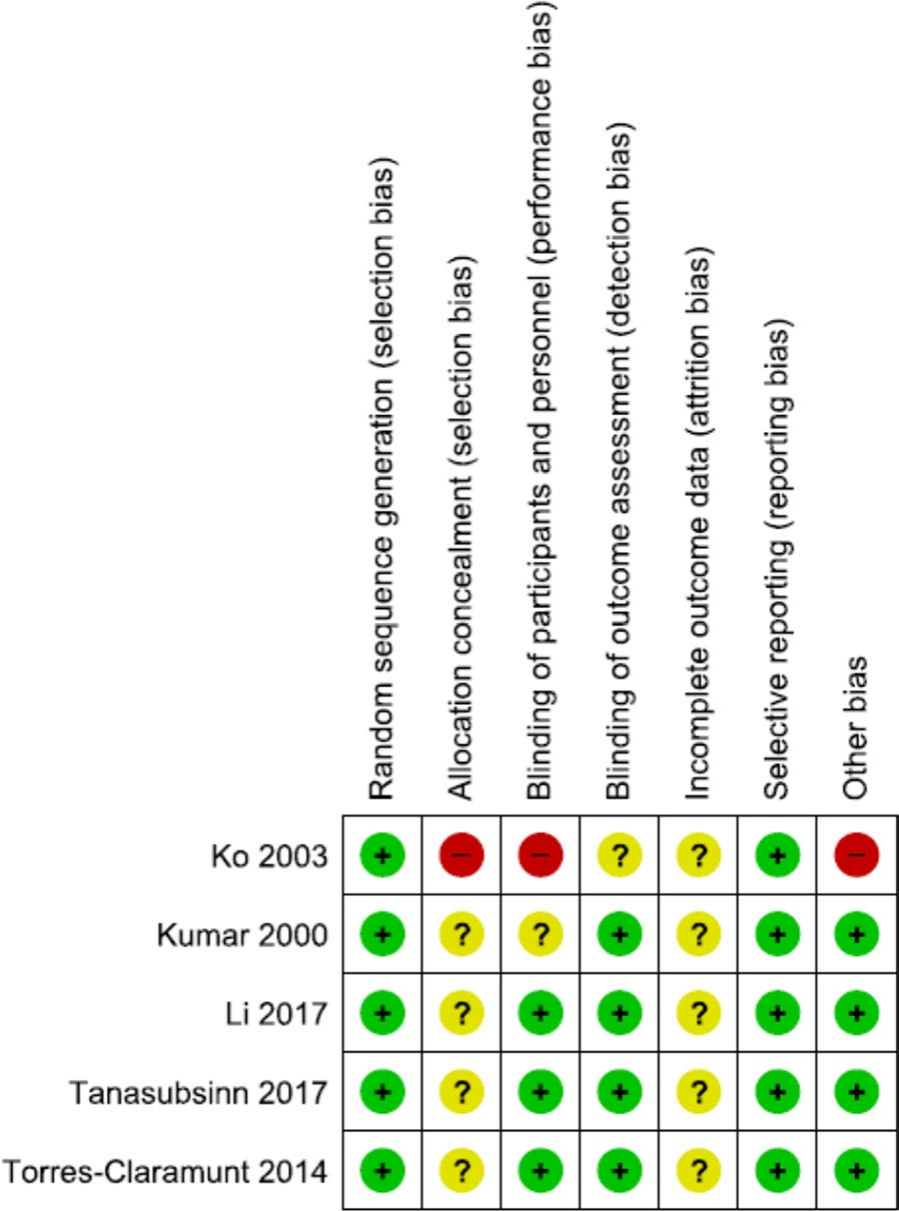
Risk of bias assessment in the included studies

The overall search with selected keywords resulted in the identification of 102 studies, of which only five studies met the inclusion criteria. Four studies used the tourniquet to achieve a bloodless field. All included studies inserted a vacuum drain into the knee and removed it within 72 h; however, no information regarding the use of tranexamic acid was described in all studies. All studies published from 2014 onward used anticoagulant for venous thromboembolism prevention. The PRISMA (Preferred Reporting Items for Systematic Reviews and Meta-Analyses) flow diagram [12] for study identification is shown in Fig. 1. The risk of bias assessments is shown in Fig. 2.

### Hemoglobin reduction post-operation (hemoglobin level differences between before and after surgery)

**Fig. 3:**
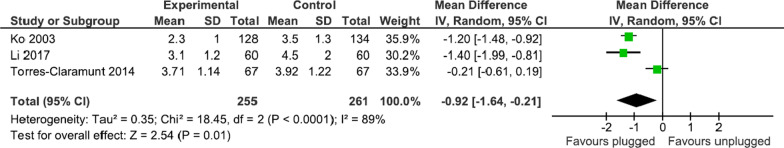
Meta-analysis of post-operative hemoglobin reduction between the plugged and unplugged groups.* CI* Confidence interval,* SD* standard deviation

Three studies reported changes in hemoglobin levels before and after surgery, involving 516 patients (255 in the plugged group and 261 in the unplugged group). The change in hemoglobin level was less in the plugged group than in the unplugged group (mean difference 0.92 g/dL; 95% CI − 1.64 to − 0.21, *p* = 0.01), as shown in Fig. 3). However, most studies did evaluate total blood loss and thus this parameter could not be analyzed.

### Total drained blood

**Fig. 4:**
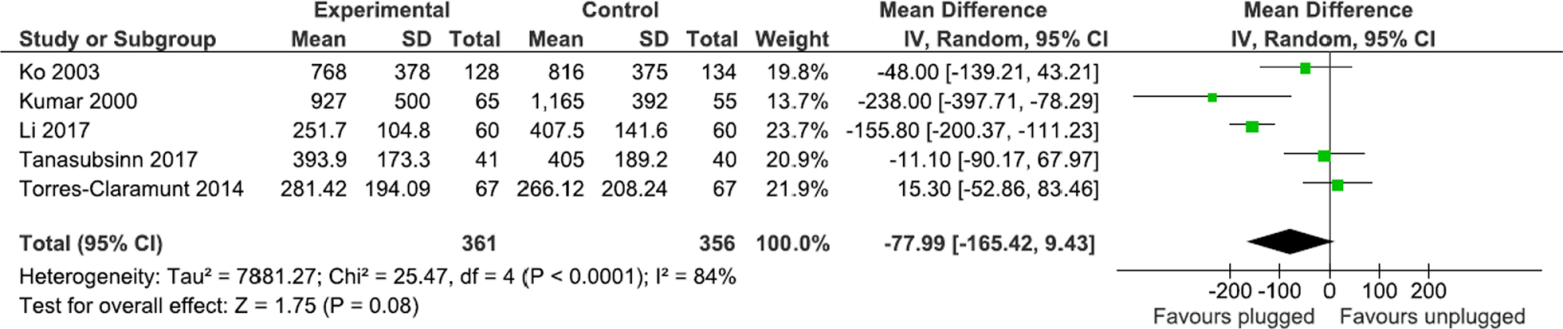
Meta-analysis of total drained blood between the plugged and unplugged groups

All five studies, involving 717 patients (361 in the plugged group and 356 in the unplugged group), reported total drained blood. The mean difference in total drained blood was lower in the plugged group than in the unplugged group (77.99 mL), but the difference was not statistically significant (95% CI − 165.42 to 9.43, *p* = 0.08) (Fig. 4).

### Drained blood at 24-h post-surgery

**Fig. 5:**
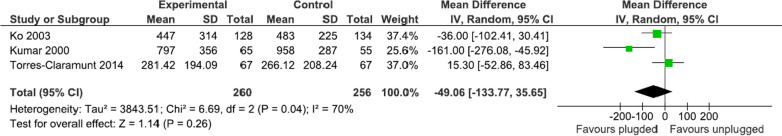
Meta-analysis of drained blood at 24 h after surgery between the plugged and unplugged groups

A total of 516 patients (260 in the plugged group and 256 in the unplugged group) were included in the analysis of drained blood at 24 h. The average difference of drained blood at 24-h post-surgery in the plugged group was 49.06 mL, which was less than in the unplugged group. However, this difference was not statistically significant (95% CI -133.77 to 35.65, *p* = 0.26) (Fig. 5).

### Blood transfusion rate

**Fig. 6:**
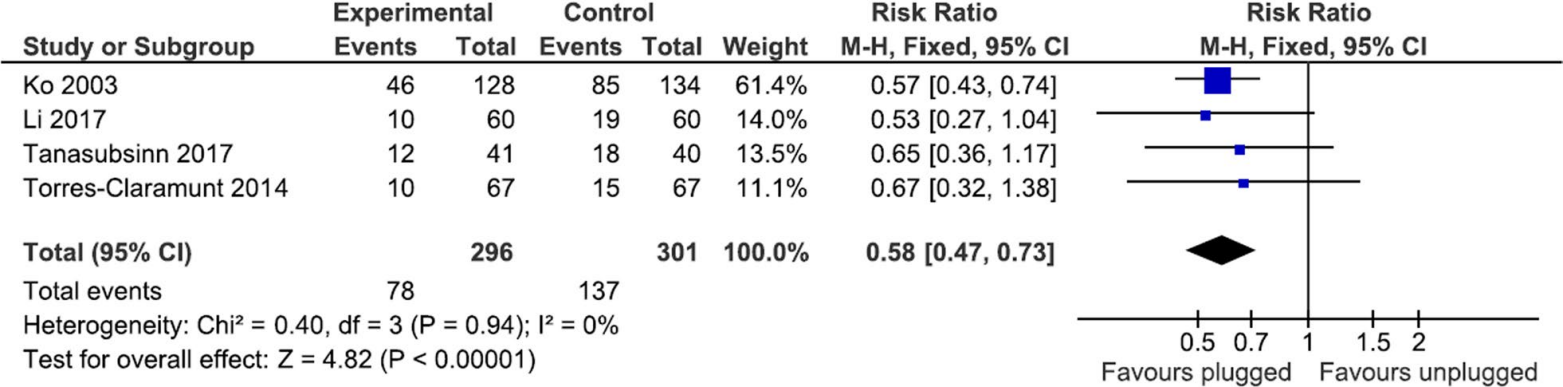
Meta-analysis of blood transfusion between the plugged and unplugged groups

Blood transfusion was reported in four studies, involving 597 participants (296 in the plugged group and 301 in the unplugged group). All studies had a similar transfusion protocol, including a hemoglobin level of < 8 g/dL or presentation of anemic symptoms). The analysis demonstrated that the plugged group had a statistically lower risk of receiving blood transfusion (risk ratio = 0.58, 95% CI 0.47–0.73, *p* < 0.00001) (Fig. 6).

## Discussion

Total knee arthroplasty is currently one of the most common orthopedic procedures performed in the elderly. Historical reports of blood loss in TKA range from 600 to 1790 mL [2, 3]. Several studies have focused on the amount of blood loss and how to minimize it. Strategies to reduce allogeneic blood transfusion in TKA include a tourniquet, autologous blood transfusion, intraoperative blood saving, and hypotensive anesthesia. Medications such as erythropoietin can help increase preoperative hemoglobin levels. Currently, tranexamic acid, with its antifibrinolytic property, has become the most widely used medication to reduce postoperative blood loss [13, 14].

Femoral canal intramedullary plug is a quick and straightforward surgical technique. However, its efficacy in reducing blood loss in TKA has never been definitively proven. While many studies have reported reduced blood loss with the use of femoral plugs, others report the reverse. The first RCT on blood loss reduction by femoral canal plug was reported by Raut and colleagues [15] in 1993. These researchers showed that the plugged group suffered significantly less blood loss than the unplugged group (mean blood loss: 752 vs. 1002 mL, respectively). However, the data in this study did not provide the standard deviation of the data, and contact with the corresponding author did not result in additional data. Therefore, we excluded this study from our meta-analysis.

Some retrospective studies comparing TKA with and without femoral canal bone plugs have been published. Protzman et al. [16] retrospectively reported 55 patients who underwent TKA, with 26 in the plugged group and 29 in the unplugged group. The mean blood loss was 960.8 mL in the plugged group and 1065.9 mL in the unplugged group, and the difference was not significant. In contrast, Batmaz et al. [17] retrospectively reviewed the outcomes of 288 patients who had TKA, 96 in the plugged group and 192 in the unplugged group. These authors found that the plugged group had a significantly higher postoperative hemoglobin level than the unplugged group (10.01 vs. 9.02 g/dL, respectively), while the preoperative hemoglobin levels were similar (12.5 vs. 12.7 g/dL). They also found that total blood loss was significantly lower in the plugged group than in the unplugged group (729.84 vs. 979.7 mL). To provide reliable results on this issue, the inclusion criteria of the present study were RCTs which compared the amount of blood loss in TKA between patients with and without femoral canal bone plugs. Also, studies without a control group, with incomplete data, or retrospective studies had to be excluded.

We chose four parameters for data analysis in our meta-analysis, namely, hemoglobin reduction, total drained blood, drained blood at 24-h post-surgery, and blood transfusion rate. Although we intended to include the total blood loss as an investigated parameter, no data were available in most of the studies. Hemoglobin reduction was chosen as the primary outcome because it correlated with the total blood loss by direct calculation [18]. In contrast, the drained blood was apparent, but did not represent total blood loss.

The resected bone piece is commonly used for the femoral canal bone plug [19]. Some surgeons also use bone cement to seal the femoral canal opening. Torres-Claramunt et al. [9] allocated TKA patients into three groups: those receiving bone plug or bone cement, and those with no bone plug. We only analyzed results from the plugged and unplugged groups to keep the protocol of all studies in the meta-analysis similar. Although some contemporary femoral component designs have closed boxes that can cover the intramedullary canal and act like a plug, all of the implants used in the included studies were used in the open box system.

The results of the present meta-analysis demonstrate that the plugged group had a significantly lower hemoglobin reduction and a lower transfusion rate than the unplugged group. However, the total drained blood and drained blood at 24 h postoperatively were similar between the two groups. These results demonstrate that significant amounts of blood loss were hidden, a finding supported by other studies which reported that the blood loss was similar in TKA with a drain or TKA without a drain.

There were a number of limitations to this meta-analysis. First, very few studies were available for analysis. Second, some studies did not mention the transfusion criteria with regard to symptoms of anemia. Lastly, the included studies did not mention other variable factors that might affect postoperative blood loss, such as anticoagulants for preventing venous thromboembolism.

## Conclusions

This meta-analysis demonstrates that a femoral canal plug could reduce postoperative blood loss and transfusion rate in unilateral primary TKA.

## Data Availability

The datasets generated in the current study are not publicly available but are available from the corresponding author.
